# Teaching Recovery Techniques: evaluation of a group intervention for unaccompanied refugee minors with symptoms of PTSD in Sweden

**DOI:** 10.1007/s00787-017-1093-9

**Published:** 2017-12-19

**Authors:** Anna Sarkadi, Kajsa Ådahl, Emma Stenvall, Richard Ssegonja, Hemrin Batti, Parthena Gavra, Karin Fängström, Raziye Salari

**Affiliations:** Child Health and Parenting (CHAP), Department of Public Health and Caring Sciences, Uppsala University, BMC, Husargatan 3, 753 27 Uppsala, Sweden

**Keywords:** Unaccompanied refugee minors, PTSD, Depression, Prevention, Teaching Recovery Technique (TRT)

## Abstract

In 2015, a total of 35,369 unaccompanied refugee minors (URMs) sought asylum in Sweden. In a previous study of 208 URMs, we found that 76% screened positive for PTSD. This study aimed to (1) evaluate the indicated prevention program Teaching Recovery Techniques (TRT) in a community setting and describe the program’s effects on symptoms of PTSD and depression in URMs; and (2) examine participants’ experiences of the program. The study included 10 groups. Methods for evaluation included the Children’s Revised Impact of Event Scale (CRIES-8) and the Montgomery–Åsberg Depression Rating Scale Self-report (MADRS-S) at baseline and at post-intervention. Qualitative interviews were conducted with 22 participating URMs to elicit their experiences. Pre- and post-measures were available for 46 participants. At baseline, 83% of the participants reported moderate or severe depression and 48% suicidal ideation or plans. Although more than half (62%) of the participants reported negative life events during the study period, both PTSD (CRIES-8) and depression (MADRS-S) symptoms decreased significantly after the intervention (*p* = 0.017, 95% CI − 5.55; − 0.58; and *p* < 0.001, 95% CI − 8.94; − 2.88, respectively). The qualitative content analysis resulted in six overall categories: social support, normalisation, valuable tools, comprehensibility, manageability, and meaningfulness when the youth described their experiences of the program, well reflecting TRT’s program theory. Overall, results indicate that TRT, delivered in a community setting, is a promising indicated preventive intervention for URMs with PTSD symptoms. This successful evaluation should be followed up with a controlled study.

## Introduction

In 2015, nearly 90,000 refugee children arrived in Europe without their families [1]. Sweden alone received over 35,000 (40%) of these children, followed by Germany (16%). A substantial majority of these children were males (91%), mainly aged between 16 and 17 (57%). About half (51%) were Afghans.

Unaccompanied refugee minors (URM) are the most vulnerable of all immigrant and refugee groups [2]. Many of them have experienced multiple traumas including exposure to armed conflict, displacement, loss of close relationships, loss of security, eyewitnessing violence, as well as experiencing personal violence such as physical aggression, torture, and rape [3, 4]. The asylum-seeking process with the related feelings of uncertainty about the future is an additional stressor with further mental health implications [5]. Therefore, it is not surprising that trauma-related problems such as posttraumatic stress disorder (PTSD), anxiety and depression are particularly common among URMs. For instance, a recent study in Norway and Belgium [6] showed that 53% of URMs reported high levels of PTSD symptoms which did not change over an 18-month period [7]. Another study in Norway, that followed up URMs who had received residence, indicated that even several years after resettlement, more than 40% of them continued to show high levels of PTSD symptoms [8]. Thus, PTSD seems to be both prevalent and persistent in asylum seeking children [9].

There are several theoretical frameworks with accompanying empirical studies that can be used to understand how people exposed to severe negative events can cope, enhance positive outcomes and avoid negative ones [10]. Within a resilience and mental health model, good or adaptive outcomes can include both higher levels of positive psychological and social outcomes, such as pro-social behaviour or school results, and lower levels of mental health symptoms [11]. Important factors that affect mental health outcomes are found on all levels of social context, i.e. individual, family, peer and community level. These factors or processes can be generally grouped into protective and promotive, but are not universal and vary within the sociocultural context and over time [12]. Nevertheless, it seems that interpersonal and sociocultural factors, such as parental support and monitoring, as well as community acceptance, affect outcomes more than static factors, such as parental education or school retention per se. Silove’s theory of adaptation and development after trauma is another important theory which emphasises the importance of safety, attachment, justice, identity-role, and existential meaning [13]. Importantly, Silove distinguishes between the “survival risk group” with severe impairment and the much larger “adaptive risk group”, where mental health problems, mainly PTSD, imply ongoing, but not specialised mental health service needs [14]. This latter group is or should be the focus of mental health initiatives for post-conflict societies or subgroups of migrants who arrive in host societies after armed conflict.

Finally, Antonovsky’s Sense of Coherence theory has been very useful in developing the science around coping and resilience in the face of extremely adverse events. The theory’s three dimensions have been found relevant in a wide range of sociocultural settings. On the intrapersonal level, the dimensions of meaningfulness, comprehensibility and manageability seem central to people’s ability to cope and adapt after trauma [15]. Hence, there is a need for interventions that can address the needs of the “adaptive risk group” on the community level, while taking into account our knowledge of the importance of inter- and intrapersonal promotive and protective factors. In addition, we need knowledge about the specific techniques that might be used to teach people the necessary skills for symptom reduction.

According to the NICE guidelines [16] children who suffer from posttraumatic stress symptoms should primarily be offered trauma-focused cognitive behavioural therapy (TF-CBT), adjusted to suit their age, circumstances, and level of development. However, studies that support the effectiveness of TF-CBT have mainly been conducted on sexually abused children [16–18] with studies on refuge children being scarce, especially considering the large number of affected children [19, 20]. In Sweden, the prevailing model of PTSD is long-term individual therapy which is costly and unfortunately not accessible timely or equally. At the beginning of this study, the waiting time for therapy at Red Cross Centres for trauma victims was 6–12 months in the region. Similar waiting times were reported by Child and Adolescent Psychiatry units around the country, many of whom refusing to treat children and youth while in the asylum process, due to lack of stability, claimed necessary for successful PTSD treatment. Ironically, instability is the very hallmark of many of these children’s existence. From a public health point of view, it is simply not acceptable that large groups in society suffer from mental health problems that have long-term consequences for their health and well-being, without being offered adequate and timely support.

Teaching Recovery Techniques (TRT) [21] is a manualised intervention based on TF-CBT, developed by the Children and War Foundation in Norway. It was explicitly built to meet the needs of low-resource settings where large numbers of children needed intervention. TF-CBT includes both stress-management skills that help children to better process their trauma-related emotions and thoughts, as well as gradual exposure to traumatic experiences that assist children to gain mastery over trauma reminders. TF-CBT intervention has some common components [22, 23]. Psychoeducation about PTSD symptoms helps to normalise children’s reactions to traumatic experiences. Parenting skills give parents the necessary tools to ensure a positive parent–child relationship. Relaxation skills such as controlled breathing help children alleviate the stress-related physiological changes. Affective modulation skills such as positive self-talk and positive imagery encourage children to express and regulate their feelings and emotions. Cognitive coping and processing, i.e. recognising the interrelation between thoughts, feelings, and behaviours and offering ways to change inaccurate and unhelpful thoughts promote better affective regulation. Trauma narrative helps children to correct their cognitive distortions about these experiences, and reduces their negative impact. In vivo mastery of trauma reminders helps children to overcome their avoidance behaviour. Conjoint child–parent sessions help facilitating parent child communications about children’s traumatic experiences and addressing other family issues. Finally, enhancing future safety and development prevents future trauma and helps children return to a normal developmental trajectory. The blend and dosage of components used in each TF-CBT intervention varies depending on the target population and type of trauma.

TRT is a group intervention based on the principles of TF-CBT that includes five sessions for young people and two for their caregivers/guardians. The sessions for children incorporate the following components of TF-CBT: psychoeducation, relation skills, affective modulation skills, cognitive coping and processing, trauma narrative, in vivo mastery of trauma reminders and enhancing future safety and development. Therefore, TRT enables normalisation of reactions to trauma, offers children emotional support, and provides them with strategies to cope with intrusive thoughts and memories, regulate their arousal, and expose themselves to avoided thoughts and situations. These strategies and techniques are modelled and practiced during the sessions. The two sessions for caregivers are held without the children. Caregivers are provided with psychoeducation about traumas and receive information about what children learn during their sessions and how caregivers can help youth to cope with past and ongoing traumas. It is hypothesised that by learning and using the coping strategies presented in TRT, children are able to assume control over their minds and bodies and therefore experience a reduction in their PTSD and depression symptoms. Normalisation is assumed to relieve children from shame and fear, whereas the safe environment provided by caring adults is geared to rebuild youth’s trust in the adult world and provide social support. The intervention can be provided by staff without previous therapeutic experience, but requires a 3-day structured training program where group leaders learn how to use the manual.

TRT has been tested in a number of settings. High acceptability and large-to-moderate effect sizes were reported for posttraumatic stress measures, depression and grief in Palestine and Gaza [24, 25] and in Thailand after the tsunami [26]. Another study in Gaza reported a modest reduction in PTSD symptoms and no change in depression [27]. The authors argued that the modest effects might be due to the imminent threat of war and the high level of ongoing stress. A study in the UK on refugee youth demonstrated short-term effects, that were not maintained at the follow-up [28]. Implementation problems, high rates of dropout at follow-up, as well as instability were reported as possible explanations for the long-term null findings in this study. In sum, there is some evidence to support the usefulness of TRT in youths with PTSD symptoms, but studies in the Scandinavian context and on the target group of URMs are needed.

The current study aimed to [1] evaluate the effectiveness of TRT in reducing the symptoms of PTSD and depression in URMs when offered in a community setting in Sweden; and [2] examine participants’ experiences of the program based on theories of posttraumatic adaptation.

## Methods

### Participants

**Fig. 1:**
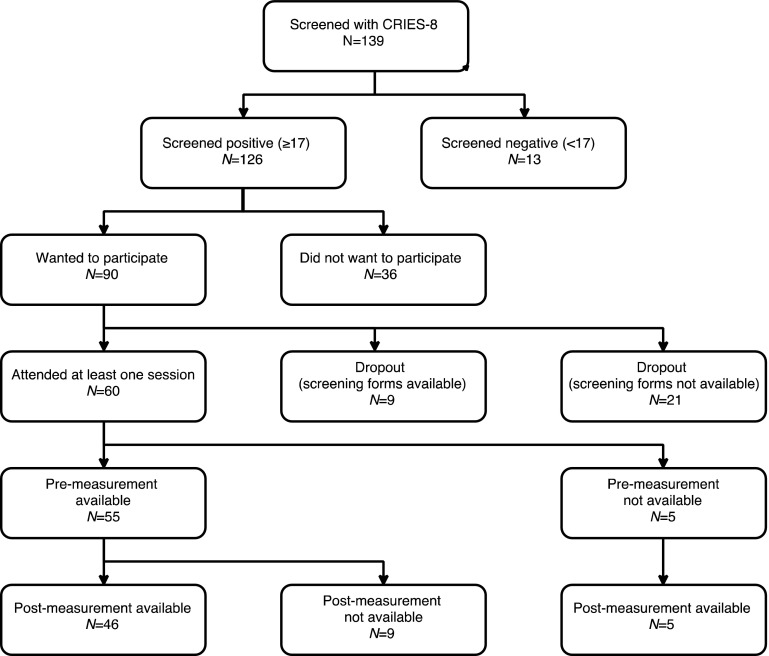
Flowchart of participants

The intervention was offered to the URMs who met the following inclusion criteria: were between 13 and 18 years old, scored 17 or more on the Children’s Revised Impact of Event Scale (CRIES-8) [29], and could nominate a person over 18 years old who agreed to participate in the TRT sessions for caregivers. Figure 1 shows the participants flow in the study. Out of 139 URMs who were screened, 126 (91%) screened positive for PTSD symptoms and of those who screened positive, 90 (71%) expressed interest to participate in the intervention. Of these 90, screening forms for later analysis were available for 69 youth. A total of 60 of 90 (67%) URMs attended at least one session, indicating a dropout rate of 33% before the intervention. Of the 60 youths attending at least one session, five lacked baseline measures (in one of the groups, group leaders had collected post-measurements only) and nine did not complete the post-measurement. Thus, 46 (77%) out of 60 children who started the intervention had both pre- and post-measurements and were included in the main analyses (84% of those with baseline measures had post-measures as well). The sample included 43 boys and 3 girls between 14 and 18 years of age (mean = 16.13). The vast majority of children were from Middle East with Afghans constituting the largest group followed by Syrians. Characteristics of these children are presented in Table 1.

**Table 1:** Baseline characteristics of participants (*n* = 46)

Variable	Mean (SD)
Age (years)	16.13 (0.96)
CRIES-8 screening	30.02 (5.51)
CRIES-8 pre-test	29.02 (6.33)
MADRS-S pre-test	29.27 (10.34)

	% (*n*)
Gender (boys)	93.5 (43/46)
Suicidal ideation (pre-test)	47.8 (22/46)
Depressed (pre-test)	82.6 (38/46)

### Procedure

Recruitment of URMs and delivery of TRT was done in the following settings:

1. An asylum health care centre—two of the nurses working in this centre were trained to deliver TRT;
2. A Red Cross Treatment Centre for Trauma—the centre maintained a waitlist and the intervention was offered to youth on this list;
3. School health services—the municipality had educated staff that could offer the intervention;
4. Group homes for URMs—the social services in three municipalities had trained some of their staff in TRT.

Recruitment was conducted by the members of the research team or the local personnel trained in TRT. The information was given in Swedish. Professional interpreters were present during the sessions and repeated all the oral information in the children’s language. They were also available to assist children in reading the screening and informed consent forms.

Youth were informed about the study and the eligibility criteria and also that participating in the study was voluntary and did not have any impact on their possibility to receive other services or on their asylum-seeking process. Then, the Children’s Revised Impact of Event Scale (CRIES-8) was handed out. Those with scores ≥ 17 were eligible to attend the intervention and received the information sheet about the study and were asked to sign the consent form if they were interested to attend the intervention.

All children who screened positive and expressed interest to participate were offered the intervention. They were asked to come in earlier for the first session to complete the pre-measures before the intervention started. Post-measures were collected in a separate session that took place two weeks after the final intervention session (first follow-up). Three to six months after the intervention, children were invited to a second follow-up session to complete the same measures again. At both follow-up sessions, youth completed the questionnaires first and then received a repetition of the techniques learned during the five sessions of the program. The second follow-up was finished with the qualitative interviews.

### Intervention

Teaching Recovery Techniques (TRT) includes a manual, outlining the theoretical background and describing the content of each session in detail. In addition, there is a workbook for practitioners that provides some examples on how the content of each session can be presented. Both the manual and the workbook were translated to Swedish before the first training for the practitioners took place.

TRT includes five sessions for young people and two for their caregivers. Each session lasts between 90 and 120 min. For children, sessions one and two focus on intrusion, session three is about arousal and sessions four and five deal with exposure. All the sessions include active skills training such as modelling, rehearsal and homework. The first session starts with some psychoeducation aimed to normalise reactions to traumatic events. Then, the “safe place” technique is introduced and practiced to regulate arousal. In session two, youth learn about imagery techniques, dual attention tasks (following the same principles as Eye Movement Desensitization and Reprocessing—EMDR), dreamwork and distraction. The third session includes presenting and practicing the following techniques to deal with arousal: relaxation, breath control, and coping self-statements (affective modulation). In addition, participants learn about the “fear thermometer” (which is a subjective unit of distress measure), activity scheduling, and sleep hygiene. In session four, reminders of traumas are discussed, the concept of grading and personalised fear hierarchy is revisited, and children are given time to plan for a real-life graded exposure practice. In session five, participants learn how they can expose themselves to traumatic memories by drawing, writing and talking while using the previously learned coping techniques such as imagery techniques, relaxation, and positive self-talk. The session finishes with discussions about looking to the future.

The program also includes two sessions for caregivers. These sessions are held without the children. The first session includes psychoeducation about traumas and how they impact children and adults. In session two, caregivers are presented with information about what children learn during their sessions and how caregivers can help youth to cope with past and ongoing traumas. The first caregivers’ session is recommended to be held before the first session for children while the second caregivers’ session is recommended to be delivered sometime between sessions one and three for the youth.

### Outcome measures

For both outcomes of interest in this project, more than one well-validated measure was available. We selected the following measures based on (a) their psychometric properties; (b) their availability in Swedish; (c) their availability in other languages commonly used by the target refugee population in Sweden; (d) their potential to be used later in the routine practice (e.g. ease of use, brevity, and cost).

Children’s Revised Impact of Event Scale (CRIES-8) [29] is a brief self-report measure of PTSD symptoms including four items related to intrusion and four items related to avoidance. The CRIES has been used in most previous trials of TRT. The total score, obtained by summing up all the eight items, has shown to have good internal consistency, and to successfully categorise over 75% of children with and without a PTSD diagnosis [29, 30]. In our own pilot study in Uppsala [31], the internal consistency for the total score was good (*α* = 0.75) and the factor structure of the scale was confirmed.

The Montgomery–Åsberg Depression Rating Scale Self-report (MADRS-S) [32] is a 9-item scale widely used in primary care in Sweden. Guidelines in our safety protocol were based on question nine, assessing suicidal ideation and intention.

We also asked the youth about life events. The questions concerned the areas of family, friends, school, housing, and migration process. Each area contained positive and negative statements. Examples of negative and positive items were: “I have been notified by the Migration Board that my asylum application has been rejected/granted”; and “I have moved school and I like my new school better/worse”.

The quantitative outcome measures were collected at baseline, at the first follow-up session which took place two weeks after the final group session (i.e. six weeks post-baseline) and at the second follow-up session which took place 3–6 months after the completion of the intervention. Qualitative interviews were conducted with 22 youth from seven groups in the format of focus group interviews, at the second follow-up. We used an interview guide (Table 2) to increase the credibility of the study. The interview questions were based on the theories of Antonovsky and Silove [13, 15] who have contributed with knowledge about factors that promote coping with traumatic events. Thus, we wanted to know whether the participants had experienced social support, normalisation, and safety in the group, whether they had increased their understanding of PTSD symptoms (comprehensibility), learnt skills and use them for coping with their posttraumatic stress symptoms (manageability and meaningfulness), and how they felt about their participation in the group (engagement) and about talking about the group with others (identity creation). We also wanted to know more broadly about their experiences with the program.

**Table 2:** Interview guide

The interview will be recorded. No personal data will be linked with the recorded material. Is that ok? Then let’s start.
1. How has it like to participate in the group?
What has been important?
Would you have liked more/less of something?
2. How has it felt like to hear others tell their stories?
3. What have you learned about how people can react after traumatic experiences/events?
4. What can you do when you feel stressed, scared, have trouble sleeping or you are thinking of the things you have experienced? Describe.
5. Did you feel comfortable in the group? Explain, what made you feel this way?
6. Can you tell me about the relationships in the group?
Have you gained some new friends within the group?
Do you meet them outside the group sessions?
7. Would you recommend the group to someone your age? Why?
What would you say?

An interpreter was present at all interviews, that were conducted by a master student (P.G.). Group leaders were sometimes present, but did not actively participate in the focus group discussion. We chose the focus group format because we were interested in hearing participants discuss their experiences with the same group that had shared the experience of TRT, explicitly interested in the role of the group in the outcomes described. After about five groups, there was a perceived saturation of data, but we wanted to collect data from all groups that had been planned for follow-up. Having 22 interview subjects is regarded as a rather high number in qualitative studies, especially considering the vulnerable nature of this target group.

### Data analyses

#### Quantitative data analysis

In this paper, we only focused on the pre- and post-measures as the 3- to 6-month follow-up was still ongoing.

We assessed the intervention effectiveness by examining [1] within-subject changes from pre- and post-intervention using the one-sample *t* test, and [2] the proportion of subjects that improved compared to the expected natural remission using the exact binomial test. The PTSD symptoms prevalence rates presented in Tam et al. [9] and the depression symptoms prevalence rates presented in Vervliet et al. [6] were used to derive the remission rates which in turn were converted into remission probabilities over the 6-week treatment period using the expression of probability = 1 − e^(−rate × time)^. A sensitivity analysis assuming double the remission rates was also conducted. We also examined the associations between improvement status and the variables hypothesised to influence it (i.e. suicidal ideation, program attendance, and type of housing facility) using logistic regression analyses.

To provide a picture of the clinical significance of change in symptoms of PTSD and depression following TRT, we classified the participants as recovered, improved, unchanged or deteriorated based on the Reliable Change Index (RCI) and Clinically Significant Change (CSC) approach [33, 34]. This approach incorporates both a measure of whether the change in scores is larger than what is expected due to measurement error of the tool (statistical significance) as well as the participant’s shift from a clinical state to a non-clinical state (clinical significance). The analyses were conducted using R version 3.2.5.

#### Qualitative data analysis

The interviews were analysed using a mix of deductive and inductive analysis, following the steps of content analysis, as described by Graneheim and Lundman [35]. The first phase was the inductive part of the analysis where the data were used to identify emerging themes according to the following process. During the manifest phase, the text was first divided into meaning units, signifying text units that relate to each other in content. The next step was condensation, a process for shortening sentences while preserving the essence of the content. Then, the condensates were assigned codes, which involved the analysis of the latent content of the category at hand. A code resulting from latent content analysis is essentially a shorthand for a number of related meaning units and their underlying meaning. Thus, the condensate “hearing others share experiences made me understand that I am not alone with my problems” became the code “normalisation of experiences”. The empirically derived codes were then tested against the theory-driven categories social support, normalisation, safety, comprehensibility, manageability, meaningfulness, engagement, and identity creation and theory-driven categories that did not match with empirically driven categories were excluded. In the final stage of the analysis, the inductive and deductive material were merged and the final categories were designated.

### Ethics

The project received clearance from the Regional Ethics Committee (Dnr 2016/348). In vulnerable populations, it is especially important that participants understand that research is voluntary and their access to services (or in refugee populations their possibility to stay in the country) will not be affected by their participation. Therefore, apart from written consent at the beginning of the study, we made sure that situational consent was received for the interviews.

According to Swedish legislation, minors who are 15 years or older and capable to do so are entitled to decide on their own research participation. Those under 15 need the consent of their legal guardians. However, information was provided to all legal guardians, regardless of URM’s age to ensure good collaboration and reduce the risk of self-harm by ensuring the possibility of information sharing. To reduce risks, a special safety protocol was developed and used during the study, providing guidelines to practitioners when to contact specialised services. A routine was established for when this had to be done immediately, through contacting emergency child psychiatry services.

## Results

### Attrition analysis

Out of the 69 of eligible URMs who had expressed interest in participating in TRT and their screening forms were available, nine did not start the intervention and five who started the intervention did not complete the pre-intervention measures and therefore could not be included in the analyses. An attrition analysis showed no statistically significant differences between these 14 children and the 55 who attended at least one session and completed the pre-intervention forms, with respect to age and CRIES-8 screening scores. Of the 55 children who started the intervention and completed the pre-intervention measures, nine did not complete the post-intervention forms. Again, there were no statistically significant differences between the two groups (9 without and 46 with post-intervention measures) with respect to age, CRIES-8 and MADRS-S pre-intervention scores.

### Intervention attendance

The attendance rate was high for the 46 URMs included in the analyses (*M* = 4.20, and SD = 0.88 sessions): one URM (2.1%) attended one session only, eight (17.4%) attended three sessions, 17 (37.0%) attended four sessions and 20 (43.5%) attended all the five sessions.

### Intervention effectiveness

The one-sample *t* test revealed a statistically significant reduction in both PTSD and depressive symptoms from pre- to post-intervention (Table 3).

**Table 3:** Differences in PTSD and depression symptoms at pre- and post-intervention (*n* = 46)

Variable	Pre-test mean (SD)	Post-test mean (SD)	Dependent *t* test	*p* value
CRIES-8	29.02 (6.33)	25.93 (5.96)	− 2.49	0.017
MADRS-S	29.26 (10.34)	23.39 (10.55)	− 3.93	0.001

The natural remission for PTSD symptoms in URMs was estimated to be 1.1% over the 6-week treatment period based on 10% change in the 1-year prevalence rate reported by Tam et al. [9]. Similarly, the natural remission rate for depressive symptoms in URMs was estimated to be 0.12% over the 6-week period based on the 0.5% 6 months prevalence change reported by Vervliet et al. [6]. The exact binomial test showed that the likelihood of improvement given the treatment was significant for both PTSD (success = 26, *n* = 46 trials, probability = 0.011, *p* < 0.001) and depression symptoms (success = 35, *n* = 46 trials, probability = 0.0012, *p* < 0.001). Doubling the remission rates as a sensitivity analysis, did not change the results.

The logistic regression analysis (Table 4) showed that for PTSD symptoms, a high level of PTSD symptoms at the commencement of the intervention was significantly related to larger symptom reduction at post-intervention (OR 1.31, 95% CI (1.13, 1.48), *p* = 0.003). None of the other variables, i.e. housing, program attendance and suicide ideation were significantly related to improvement of PTSD symptoms. For depressive symptoms, URMs with suicidal ideation were approximately 10 times more likely to report reduced depression symptoms following the intervention compared to URMs without suicidal ideation [OR 9.91, 95% CI (7.71, 12.11), *p* = 0.041]. None of the other variables were significantly related to reduced depression.

**Table 4:** Logistic regression analysis investigating improvements on CRIES-8 and MADRS-S in relation to pre-test scores, TRT attendance, housing and suicidal ideation

Adjusted ORs output variable	Estimate	SE	*p*	OR (95% CI)
CRIES-8				
CRIES-8 pre-test	0.27	0.09	0.003**	1.31 (1.13, 1.48)
TRT attendance	− 1.15	0.94	0.219	0.32 (− 1.52, 2.15)
Housing	0.18	0.77	0.811	1.20 (− 0.31, 2.71)
Suicidal ideation	− 0.74	0.83	0.374	0.48 (− 1.16, 2.11)
MADRS-S				
MADRS-S pre-test	− 0.03	0.04	0.574	0.97 (0.88, 1.07)
TRT attendance	− 0.64	1.00	0.524	0.53 (− 1.44, 2.49)
Housing	− 1.07	0.84	0.203	0.34 (− 1.31, 2.00)
Suicidal ideation	2.29	1.12	0.041*	9.91 (7.71, 12.11)

* *p* < 0.05; ** *p* < 0.01

The RCI and CSC analyses showed that 22% of the participants were classified as recovered on PTSD symptoms and 33% on depression symptoms (Table 5). A few adolescents improved or deteriorated, whereas the majority were unchanged (63 and 61% for PTSD symptoms and depression, respectively) according to these indices.

**Table 5:** Number of participants in each category at post-intervention using Reliable Change Index and Clinical Significance Change approach (*n* = 46)

Category	PTSD symptoms (CRIES-8)		Depression symptoms (MADRS-S)	
	*n*	%	*n*	%
Recovered	10	21.7	15	32.6
Improved	3	6.5	1	2.2
Unchanged	29	63.1	28	60.9
Deteriorated	4	8.7	2	4.3

A total of 62% of the youths reported negative life events during the program, most often a friend receiving refusal of asylum. However, 53% also reported positive events, such as finding new friends, moving to a better school or receiving a better placement.

### Interview results

The following codes resulted from the inductive analysis: supported by the group, normalisation of experiences, valuable tools for daily life, making sense of what happened, managing my feelings/having control, and finding meaning/looking to the future. These empirically derived codes were then tested against the theory-driven categories social support, normalisation, safety, comprehensibility, manageability, meaningfulness, engagement, and identity creation. The theory-driven categories safety, engagement, and identity creation were excluded as the data did not match with these. Valuable tools for daily life emerged instead as a new category. In the final stage of the analysis, merging the inductive and deductive material, the final categories were designated, according to the following. Supported by the group was converted to social support, normalisation of experiences to normalisation, making sense of what happened to comprehensibility, managing my feelings/having control to manageability, and finding meaning/looking to the future to meaningfulness. The final analysis process thus resulted in six overall categories: social support, normalisation, valuable tools, comprehensibility, manageability, and meaningfulness. Quotes were then selected to demonstrate each category, including negative case analysis where we explicitly looked for examples where the data did not match the overall direction of the category presented.

#### Social support

The participants expressed an appreciation for the feeling of community in the groups. Some described a trust in the group and the fact that it felt safe to open up and “ease the heart”.

*“We were so comfortable in the group, we could talk to each other like a friend, like a brother. It was very nice in this group, and it was right …”*

Another participant described that he felt nervous at the first group meeting, but then eased into the setting. Others mentioned that they would have liked more individual conversations with the group facilitators.

#### Normalisation

The opportunity to meet others in the same situation was described as valuable. The majority of participants described decreased loneliness to which having common experiences and resulting problems contributed substantially.

“*You thought you were alone with these thoughts but then when you got into the group and saw the others, you felt, yes, but now it feels a bit easier because I’m not alone with this problem.”*

Even if TRT does not explicitly encourage narratives, some of the boys told their stories to the group. A painful aspect of listening to others’ experiences was described by one of the boys:

*“When you sit and listen to the others, you are reminded of your own difficult experiences so it may feel bad. But it also helps, in some way, it takes away the edge, and you become accustomed to this hard stuff.”*

#### Valuable tools

In general, participants expressed gratefulness for having gained access to all the techniques/tools in the program.

*“These techniques we’ve learned have been good. The calmness you felt after doing these exercises, it was great to be able to feel this calm.”*

Exercises such as muscle relaxation, breath control, a “safe inner place” (arousal control exercise), and tools, such as set time for thoughts/concerns and “patting the knees” while working with a difficult image (bilateral stimulation) were most appreciated.

Participants’ experiences about what has helped with regard to symptoms and techniques differed. Some young people reported having been helped with sleeping difficulties, intrusive memories, depressed thoughts, fear and irritation.

*“… After doing exercises I’ve learned here, I’ve reduced sleeping pills.”*

Although useful for sleep problems for some, several participants stated that the techniques did not affect their sleep. Others again described the tools becoming useful some time after the group had ended.

*“Just when I joined the group, I might not have used all the methods I was taught, but afterwards, when I got into a more difficult situation, it helped me, what I learned helped.”*

#### Comprehensibility

Making sense of their past experiences and current feelings were important elements during participation, according to the youth. Accepting that past experiences cannot be undone and relating to them as an important part of one’s personal history was apparent in the youth’s responses.

*“The things that have happened to us are never forgotten. The others do not have our problems. Other people, they are more free.”*

Understanding the way their past experiences affected the youth was also discussed in the interviews. They underscored that while their experiences might have been similar, there were also differences in the problems that arose for each individual.

*“And so you have different problems. There is a problem, but it’s a bit different for everyone, it’s not the same.”*

Finally, there was mention of how these young people with their experiences fit into the new world they had come to.

*“From the first time I came to Sweden, I feel that I am a newborn, now I belong to this world as well.”*

Thus, incorporating past experiences into who they are today, seeing the similarities as well as differences in the problems presented by their peers and themselves, and understanding oneself in relation to the majority culture were all important elements of comprehensibility in this study.

#### Manageability

The term “control” was mentioned as an important factor in all of the interviews.

*“When I came to this group I had no control of myself, because everything I had been through affected me and was controlling me. The group helped me to gain control over myself.”*

The issue of control came up also when there was a perceived lack of it.

*“I can control stress and I can control my fear, I can control that I cannot sleep, but I have difficulty controlling my anger. That’s really difficult to control.”*

The participants talked eloquently about how the tools they learned helped them manage their everyday lives and symptoms.

*“And one night, I had so much trouble breathing so they had to take me to the emergency room for help. And therefore, I say that these exercises help me a lot to handle my fear and anxiety. I get these attacks every night and I think if I had not done these exercises I would have been even worse.”*

Thus, it was not a complete lack of symptoms, but their manageability that was the focus of the youth’s narratives.

*“For me the most important thing was that when you are down or you are in a mental crisis, how to get yourself out of it, how to save yourself somehow.”*

#### Meaningfulness

The youth struggled to find meaning in their experiences and how that might affect their lookout for the future.

*“The most difficult thing one ever can do is to leave their family and move elsewhere and not be able to meet them. We have left this behind us so I think we can handle whatever happens.”*

Many of the boys expressed dejection and described how, for example, current incidents in the home country really affected them. One of the young people stated that nothing can be done about the situation except to “endure”. Other useful strategies mentioned were distraction, such as listening to music, or activation, such as hanging out with friends or talking to someone they trusted.

Another participant discussed how symptoms, such as loss of appetite and insomnia were difficult to safeguard against, but could be dealt with, over time. Thus, accepting ‘what is’ and coping in face of past hurtful events and current worries was described. In addition, participants felt it was meaningful to take part in the program:

*“I would really recommend other people who are in the same situation as us, as we were then, to come here and get some tools to help themselves feel better.”*

## Discussion

Significant differences between pre- and post-measures on depressive and PTSD symptoms were seen. The analyses of reliable clinical change showed that 33% of the participants recovered based on depression symptoms and 22% recovered based on PTSD symptoms. These findings occurred despite 62% of participants experiencing negative life events during the six weeks of the program, and being in the middle of their asylum-seeking process. The qualitative interviews resulted in six overall categories: social support, normalisation, valuable tools, comprehensibility, manageability, and meaningfulness.

Our results are in accordance with the program theory of TRT, where sharing experiences in a safe and supportive environment and learning tools for coping, including trauma-specific exposure and behavioural activation, are expected to increase youth’s sense of coherence and decrease symptom burden of depression and PTSD. Specifically, we found through our qualitative interviews that the TRT program components enabled normalisation of reactions to trauma (normalisation), offered participants emotional support (social support), provided them with strategies to cope with intrusive thoughts and memories (valuable tools), regulate their arousal (manageability), and expose themselves to avoided thoughts and situations (manageability). In addition, Antonovsky’s dimensions of meaningfulness and comprehensibility clearly appeared in the narratives of youth during the interviews. In terms of Silove’s theory of adaptation and development after trauma, our participants belonged to the “adaptive risk group” where mental health problems, mainly PTSD and depression meant they needed help, even though they seemed to function to a certain extent in their everyday lives [14].

Posttraumatic stress symptoms significantly decreased from pre- to post-assessment which is in line with previous research investigating the effects of TRT [36] and other TF-CBT programs [37] in traumatised children. As we did not have a control group, we conducted analyses comparing the natural remission seen in previous publications to the observed outcomes in this study. Even when assuming double the natural remission rate calculated for a 6-week period observed in previous studies, our results show a significant likelihood of the program having an effect compared to natural remission. Our results are also in accordance with those reported by Berkowitz et al. [38] on an individual intervention for children with risk of developing PTSD, the Child and Family Traumatic Stress Intervention. This intervention is very similar to TRT, but allows greater individual adjustment and has more additional specific focus on depressive symptoms and withdrawal. Even without a focus on depressive symptoms and although not the primary target of TRT, symptoms of depression significantly decreased from pre- to post-assessment. This is also in line with previous research on TRT after the Athens earthquake [36]. Reasons why TRT might have had a positive effect on depressive symptoms include strategies, such as the “safe inner place” (arousal control) technique, and tools, such as set time for thoughts/concerns, that might have promoted youth to break the vicious cycle of negative thoughts and feelings and being out of control. In addition, getting involved in more activities and activating support networks by talking to trusted people when feeling sad might also have contributed to less depressive symptoms. Some youth reported better sleep as a consequence of relaxation techniques, and better sleep can clearly contribute to symptom improvement in depression. Finally, the presence of meaningfulness described by several participants might have contributed to improved depressive symptoms: meaningfulness is the very contradiction of the feeling of everything being meaningless, at the core of depression. The qualitative interviews confirmed the program theory regarding the use of the group setting as a safe environment to normalise experiences and contribute to meaning creation in addition to behavioural activation components.

Our subgroup analyses indicate that suicidal ideation at baseline markedly increased the odds for symptom reduction. This is all the more interesting as we had an initial ethical discussion whether we should include youth with such serious symptom burden in this light-touch intervention. The reason we decided to do so was twofold. There was no other viable option for help, given waiting times of 6–12 months to regular services that not even always took on youth during the asylum process, referring to the instability of their situation. The other reason was the advice of our international advisory board, namely that a structured program with established safety protocols and a supportive climate, although conducted by personnel outside child psychiatry services, had potential benefits, widely offsetting potential risks, given the current situation. Although we did not find empirical support in the qualitative interviews for the categories of safety and identity creation stemming from Silove’s theory, it is quite plausible that creating social support through the groups addressed needs related to the category of safety/security. Indeed, Silove recommends social and family support as a means to provide safety and security to the group who shows an adaptive response, albeit with symptoms. Similarly, Silove proposes terminating isolation, skills development, and offering counselling as a means to enhance role identity. Thus, our findings of reduction in both depressive and PTSD symptoms are well in line with the program theory of TRT and what we know about factors that affect posttraumatic adaptation and growth from other theories.

Yet, although caution in interpreting results from this observational study is required, the reduction of PTSD symptoms is remarkable considering the context in which the youths were living. First, they were enduring an ongoing asylum process; the majority of them had been in Sweden since 2015 and still did not know whether they would be granted asylum. Second, more than half of the children (62%) had been exposed to new stressful life events during the time of the intervention, something which probably added to their mental health burden. Additionally, parental support has in previous studies been shown to mitigate the consequences of trauma [9], but for unaccompanied refugee minors no parental support was available (many had no contact at all with their parents) and we do not know to what extent they were supported by caring adults to handle their new stressful experiences. For trauma-focused CBT to have optimal effect, regular practicing and homework completion are required. Although inclusion criteria said that an adult would have to participate in the parallel sessions, the youths did not turn out to have adequate and regular support in practicing the strategies included in the program.

### Strengths and limitations

An obvious limitation of this study is its lack of a control group. We have attempted to compensate for this through conducting analyses comparing our results to natural remission observed in randomised trials and by complementing our quantitative results with qualitative interviews to tap into possible change mechanisms based on the program theory employed in TRT.

Other methodological limitations relate to measurement issues. All included measures were self-report, paper and pen measures. In our previous study, although the factor structure of the CRIES-8 was confirmed in URMs in Sweden and also found to have good internal consistency, we did note difficulties for the youth to answer some of the questions and many required assistance when filling in the questionnaires [31]. Answering questions about symptoms such as intrusive thoughts or suicidal ideation requires metacognitive ability as well as a certain level of mental health literacy. Many of the boys from Afghanistan in this study had very few years of formal schooling and also generally had low health literacy. These circumstances add some uncertainty to the measures.

A strength of the study is that it was conducted in a community setting using regular service and personnel without extra financial influx to the project. It was also conducted, during a time which was especially taxing for social and mental health services, in the aftermath of the refugee crisis in 2015. Another strength is that the majority (84%) of the 55 participants who commenced the Teaching Recovery Techniques program completed it, thus increasing the validity of our findings.

For the qualitative analysis, credibility was enhanced by using an interview guide. Dependability was increased by a clear description of the analytical process and the distinction between the inductive and deductive parts of the process. Finally, transferability was enhanced by selecting all available groups for data collection and conducting negative case analysis when selecting quotes. Transferability was limited by the attrition from the interviews; we have no information on the reasons of those who did not attend these sessions.

### Future research

A trial with a controlled design, preferably using an active control group participating in a support group, is needed to increase the level of evidence for the use of TRT in unaccompanied refugee minors with PTSD symptoms. It would also be useful with larger sample sizes allowing for subgroup analyses, so youth who will be most likely to benefit from the intervention can be identified.

## Conclusion

Significant differences between pre- and post-measures on depressive and PTSD symptoms were seen, despite 62% of participants experiencing negative life events during the program, and being in the middle of their asylum process. The qualitative interviews resulted in six overall categories: social support, normalisation, valuable tools, comprehensibility, manageability, and meaningfulness. Our results are in accordance with the program theory of TRT, where sharing experiences in a safe and supportive environment and learning tools for coping, including trauma-specific exposure and behavioural activation, are expected to increase youth’s sense of coherence and decrease symptom burden of depression and PTSD. Overall, results indicate that TRT is a promising indicated preventive intervention for URMs with PTSD symptoms. Nevertheless, this is not to say that TRT should be the treatment of choice for URM with PTSD symptoms, depression and suicidal ideation or plans. What it may be, is the first stage in a stepped care model.
